# The Effects of Brain Serotonin Deficiency on Responses to High Fat Diet in Female Mice

**DOI:** 10.3389/fnins.2021.683103

**Published:** 2021-07-02

**Authors:** Shama N. Huq, Allison K. Warner, Kerry Buckhaults, Benjamin D. Sachs

**Affiliations:** ^1^Department of Psychological and Brain Sciences, College of Liberal Arts and Sciences, Villanova University, Villanova, PA, United States; ^2^Department of Psychological and Brain Sciences, Villanova University, Villanova, PA, United States

**Keywords:** serotonin, obesity, anxiety, hippocampus, mouse model

## Abstract

Clinical studies have reported an increased risk of depression and anxiety disorders among individuals who are obese, and women are more likely than men to suffer from depression, anxiety, and obesity. However, the effects of obesity-promoting diets on depression- and anxiety-like behavior remain controversial. A recent study from our group used the tryptophan hydroxylase 2 (R439H) knock-in mouse line to evaluate the impact of genetic brain serotonin (5-HT) deficiency on behavioral responses to high fat diet (HFD) in male mice. That study indicated that chronic exposure to HFD induced pro-anxiety-like effects in the open field test and antidepressant-like effects in the forced swim test in wild-type males. Interestingly, the antidepressant-like effect of HFD, but not the anxiogenic effect, was blocked by brain 5-HT deficiency in males. The current work sought to repeat these studies in females. Our new data suggest that females are less susceptible than males to HFD-induced weight gain and HFD-induced alterations in behavior. In addition, the effects of chronic HFD on the expression of inflammation-related genes in the hippocampus were markedly different in females than we had previously reported in males, and HFD was shown to impact the expression of several inflammation-related genes in a genotype-dependent manner. Together, our findings highlight the importance of brain 5-HT and sex in regulating behavioral and molecular responses to HFD. Our results may have important implications for our understanding of the clinically observed sex differences in the consequences of obesity.

## Introduction

Major depressive disorder and anxiety disorders are highly prevalent conditions that lead to substantial global disability (Kessler et al., 2012; Ferrari et al., 2014). Although the causes of these disorders are likely complex and multifactorial, existing data indicate that obesity can increase the risk of both depression (Luppino et al., 2010; Li et al., 2017) and anxiety disorders (Gariepy et al., 2010). Importantly, clinical associations do not imply causality, and whether chronic consumption of diets that promote obesity is sufficient to promote depression- or anxiety-like behavior remains debated. Indeed, while some studies report that high fat diets (HFDs) increase depression- and anxiety-like behavior (Strekalova et al., 2015; Almeida-Suhett et al., 2017; Xu et al., 2018), others suggest that these types of highly palatable diets can induce antidepressant-like or anxiolytic effects (Maniam and Morris, 2010a,b; Finger et al., 2011). It is possible that genetic, hormonal, or other factors can influence the ways in which dietary alterations impact depression- and anxiety-like behavior, but additional research is required to identify these factors.

One of the neurochemical anomalies that has long been hypothesized to contribute to mental illness is brain serotonin (5-HT) deficiency (Coppen, 1969; Delgado, 2000). Over the past few decades, the development of genetically modified mice has allowed for testing whether low 5-HT levels cause behavioral alterations relevant to disorders such as depression and anxiety. Prior work using mice with genetic reductions in brain 5-HT have consistently reported increases in impulsivity and aggression in 5-HT-deficient animals, but 5-HT deficiency does not consistently induce depression- and anxiety-like phenotypes in the absence of environmental stressors (Fernandez and Gaspar, 2012). However, work from our lab and others has shown that brain 5-HT deficiency can impair antidepressant responses (Sachs et al., 2013a, 2015) and impact susceptibility to depression and anxiety-like behavior following stress (Gutknecht et al., 2015; Sachs et al., 2015). A recent study from our lab evaluated the effects of brain serotonin (5-HT) deficiency on HFD-induced behavioral alterations in males (Karth et al., 2019). Our data revealed that HFD increased anxiety-like behavior regardless of 5-HT levels and that HFD led to a significant antidepressant-like effect in the forced swim test (FST) in wild-type (WT) but not 5-HT-deficient mice (Karth et al., 2019). Given the known sex differences in the rates of obesity (Hedley et al., 2004), depression, and anxiety (Kessler et al., 1994) and the growing evidence that men and women are differentially sensitive to some of the negative health consequences of obesity (Audureau et al., 2016), evaluating the effects of 5-HT deficiency on HFD responses in females is critical.

To model genetically induced brain 5-HT deficiency, the current study used tryptophan hydroxylase 2 (Tph2) R439H knock-in (KI) mice, which express a partial loss-of-function mutation in the brain 5-HT synthesis enzyme, Tph2 (Beaulieu et al., 2008). Homozygous KI animals from this line exhibit 60–80% reductions in brain 5-HT levels compared to their homozygous WT littermates (Beaulieu et al., 2008; Jacobsen et al., 2012). These KI animals have been shown to exhibit increased susceptibility to anxiety- and depression-like behavior induced by stress (Sachs et al., 2015) and blunted antidepressant-like responses to selective serotonin reuptake inhibitors (and HFD) compared to WT controls (Sachs et al., 2013a, 2015; Karth et al., 2019). Prior work reported a role for glycogen synthase kinase 3β (GSK3β) in the behavioral phenotypes of Tph2KI mice (Beaulieu et al., 2008), and GSK3β has also been suggested to be involved in behavioral responses to HFD (Papazoglou et al., 2015; Wakabayashi and Kunugi, 2019). Specifically, GSK3β phosphorylation (which is inhibitory) has been shown to be negatively correlated with depression-like behavior in mice exposed to HFD for 16 weeks (Papazoglou et al., 2015), which is consistent with studies showing that the inhibition of GSK3β leads to antidepressant-like effects in mice (Kaidanovich-Beilin et al., 2004; Omata et al., 2011; Perez-Domper et al., 2017). These findings, along with clinical reports identifying increases in the activity of GSK3β in postmortem brain samples from patients with major depression (Karege et al., 2007, 2012), have led to the hypothesis that GSK3β is a critical player in the development of major depression that also plays an important role in antidepressant treatment responses (Duda et al., 2020). In addition to GSK3β, HFD-induced brain inflammation has also been suggested to contribute to HFD-induced behavioral disturbances (Dutheil et al., 2016; Wu et al., 2018). In particular, the upregulation of several pro-inflammatory cytokines in the brain, including interleukin-1β (IL-1β) (Almeida-Suhett et al., 2017) and interleukin-6 (IL-6) (Wakabayashi and Kunugi, 2019), has been implicated in murine behavioral responses to HFD. Our prior work in males revealed that brain 5-HT deficiency prevents HFD-induced inhibition of GSK3β, but it had no effect on HFD-induced increases in IL-1β expression (Karth et al., 2019). The current study examined whether low brain 5-HT impacts behavioral and molecular responses to chronic exposure to HFD in females as we previously reported in males.

## Method

### Animals

Female WT and KI animals from the Tph2R439H mouse line were generated for this study by mating mice heterozygous for the mutation of interest to produce homozygous KI animals and homozygous WT littermate controls. Mice were between 2 and 4 months of age at the time of initial HFD exposure. Toe clips were obtained from mice when they were 7 days old and were used for genotyping analysis using published PCR-based methods (Beaulieu et al., 2008). Briefly, tissue was incubated in digest buffer (100 mM Tris–HCl pH 8.5, 5 mM EDTA, 0.2% SDS and 200 mM NaCl) containing proteinase K (100 μg/ml) overnight at 55°C while shaking. Samples were centrifuged for 10 min at 20,000 × *g*, and the supernatants were added to an equal volume of isopropanol. After 15 s of vigorous shaking, the samples were again centrifuged for 10 min at 20,000 × *g*. Pellets were air dried, then redissolved in deionized water. Redissolved DNA samples were analyzed using PCR primers for Tph2 genotyping: primer 1: 5′-CACCCAATTTGCCTGCCGTAG-3′ and primer 2: 5′-GTCGCAAAACATATCACAGAACTCATTCAAGA-3′. The PCR protocol for genotyping was as follows: 90 s at 95°C, then 35 cycles of 95°C for 2 min, 63°C for 30 s and 72°C for 1 min, then 72°C for 10 min.

### Diet and Housing

Prior to the start of experimentation, all mice were fed Envigo’s Teklad Global Diet (Standard Natural Ingredient Diet: ID #2019, 19% protein, 9% fat, 3.3 kcal/g). When mice were 2–4 months of age, mice of each genotype (WT and KI) were treated with one of two diets: an HFD, Envigo’s Teklad Custom Diet (Adjusted Fat Diet: ID #95217, 18.8% protein, 39.7% fat, 4.3 kcal/g) or a standard diet (SD), Envigo’s #2019 diet. Food and water were available *ad libitum*, and HFD exposure continued for 22 weeks. Animals were housed 3–5 per cage in a temperature- and humidity-controlled room that was maintained on a 12 h light-dark cycle. Behavioral testing began during the 21st week of HFD exposure. The order of testing was as follows: novel open field test (NOF), week 21 – Tuesday; elevated plus maze (EPM), week 21 – Thursday; FST, week 22 – Tuesday. All behavioral tests were conducted during the light phase with a lighting intensity of approximately 165 lux. Mice were acclimated to the testing room for one hour prior to the start of behavioral testing.

### Novel Open Field Test

The NOF was conducted exactly as described previously (Karth et al., 2019). Locomotor activity was assessed over a 20 min period in 40 cm × 40 cm plexiglass AnyBox activity chambers (Stoelting, Wood Dale, IL, United States), with a center square measurement of 20 cm × 20 cm. The total distance traveled, the distance traveled in the center, the number of entries to the center, and the time spent in the center were all calculated using AnyMaze software (Stoelting).

### Elevated Plus Maze

For the EPM, the location and activity of mice in the apparatus were measured using AnyMaze animal tracking software exactly as described previously (Karth et al., 2019). Briefly, mice were placed into one of the closed arms of the maze, and its behavior and location was tracked for a period of 5 min using a camcorder suspended above the maze. The overall height of the EPM is 50 cm, each arm is 5 cm wide and 35 cm long, and the walls of the closed arms are 15 cm high. The time spent and distance traveled in the open arms, the closed arms, and the entire apparatus were determined for each mouse. Two mice were never detected or tracked by the computer software and were not included in the analysis. One of these mice was from the KI-SD group and the other was from the WT-SD group.

### Forced Swim Test

The FST was performed as we have previously reported (Karth et al., 2019). Briefly, mice were placed in a 4 L beaker filled with approximately 2500 ml of ∼25°C water. The behavior of each mouse was recorded for 6 min using a camcorder positioned directly above the beaker. AnyMaze software was used to measure the total distance traveled, amount of time each mouse spent immobile, latency to immobility, and the number of immobile episodes.

### Gene Expression Analysis

One week following the FST, mice were killed by cervical dislocation and decapitation, after which the brains were removed and sectioned into 1 mm thick sections using a brain matrix. Bilateral tissue punches (1.5 mm in diameter, 1 mm in thickness) were taken from the hippocampus (centered approximately ±2.5 mm ML, −3.7 mm AP, −2.5 mm DV relative to Bregma) and immediately frozen on dry ice and transferred to a −70°C freezer until further processing. RNA was isolated from the tissue samples collected from the hippocampus of the left hemisphere using the Invitrogen PureLink RNA Mini Kit (Catalog #12183018A, Thermo Fisher Scientific, Waltham, MA, United States) according to the manufacturer’s instructions. RNA was frozen at −70°C until further processing.

Reverse transcriptions were performed using the Thermo Scientific Maxima First Strand cDNA Synthesis Kit (catalog #: K1652, Thermo Fisher Scientific), according to the manufacturer’s instructions. Real-time PCR was performed as we have described previously (Karth et al., 2019) using the PowerUp Sybr Green Master Mix rt-PCR kit (catalog #: A25741, Applied Biosystems, Foster City, CA, United States) according to the manufacturer’s instructions. A StepOne Plus real-time PCR machine was used to perform all analyses. The PCR protocol was as follows: 2 min at 50°C, 10 min at 95°C, and then 40 cycles of 95°C for 3 s and 60°C for 30 s. Samples were run in duplicate, but if the replicates exhibited high variability (as defined and “flagged” by StepOne software), the entire run was repeated (again in duplicate) until the replicates had sufficiently low variability. Relative expression was determined using the ΔΔCt method. One RNA sample from the WT-SD group produced a GAPDH Ct value that was approximately three and a half standard deviations from the mean. It was determined to be an outlier and not included in the gene expression analyses. Primer sequences were selected from PrimerBank (Wang et al., 2012) and can be found in Supplementary Table 1.

### Western Blotting

Hippocampal tissue punches from the right hemisphere were processed for western blotting essentially as we have described previously (Sachs et al., 2013c). Briefly, samples were lysed in ice-cold lysis buffer (1% Triton, 1 mM EDTA, 150 mM NaCl, 20 mM Tris–HCl) with protease and phosphatase inhibitors added. A small aliquot was used for protein determinations using bicinchoninic acid (BCA) assay kits (Catalog # BPA-500, Boston Bioproducts, Ashland, MA, United States). Equal amounts of protein (∼35 μg per sample) were loaded onto TGX gels (Catalog # 4561034, Bio-Rad, Hercules, CA, United States) for electrophoresis. Proteins were then transferred to PVDF membranes (Catalog # 10026934, Bio-Rad), and western blotting was performed using the following antibodies: rabbit anti-phosphorylated GSK3β (Cell Signaling, #9323, 1:300 dilution, Danvers, MA, United States), mouse anti-total GSK3β (Cell Signaling, #9832, 1:300 dilution), and rabbit-anti-GAPDH as a loading control (Cell Signaling, # 5174, 1:300 dilution).

### Statistical Analyses

Most data were analyzed by two-way ANOVA with genotype and diet as factors using JMP15 software (SAS Institute, Cary, NC, United States). When significant genotype by diet interactions were observed, Tukey’s *post hoc* tests were run using JMP15 software. To assess the significance of diet-induced changes in weight over time in the two genotypes, SPSS25 software (IBM, Armonk, NY, United States) was used to perform a three-way repeated measures ANOVA with genotype and diet as between-subjects factors and time as the within-subjects repeated measures factor (for each of the 21 weekly timepoints). The significance threshold was set at α = 0.05.

## Results

### HFD-Induced Weight Gain

**FIGURE 1:**
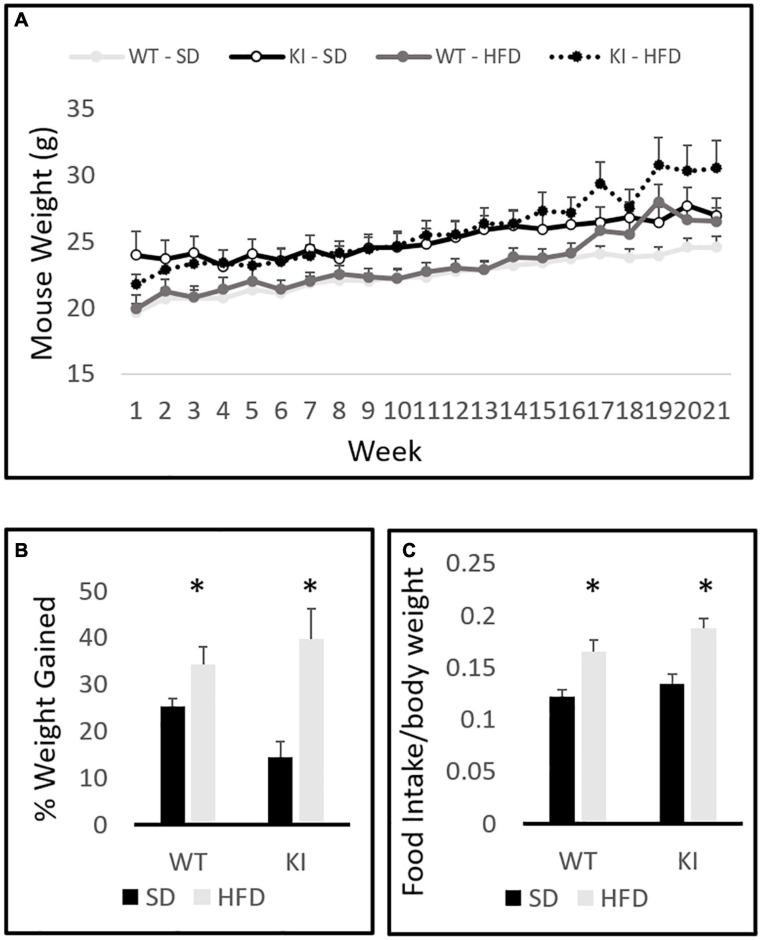
Body weights of wild-type (WT) and knock-in (KI) mice on standard diet (SD) and high fat diet (HFD). **(A)** Quantification of average body weights over the time course of the experiment. **(B)** Calculation of the average percent gain in body weight over the course of the experiment for each group. **(C)** Average daily food intake (grams of chow per gram body weight) in mice consuming either SD or HFD. Results are expressed as the mean, and error bars indicate standard error. “^∗^” indicates a significant main effect of diet by two-way ANOVA, *p* < 0.05. *N* = 10 per WT group and 11 per KI group.

The between-subjects analysis in the repeated measures diet by genotype by time three way ANOVA revealed a significant effect of diet on total weight gain [*F*_(1,41)_ = 15.51, *p* = 0.0003, Figure 1A]. A significant genotype by diet interaction was also observed [*F*_(1,41)_ = 4.8, *p* = 0.035, Figure 1A]. Tukey’s *post hoc* analysis indicated that HFD significantly increased weight gain in KI mice (*p* = 0.0004), but not in WT controls (*p* = 0.626). Within-subjects repeated measures analysis revealed a significant effect of time [*F*_(20,21)_ = 88.71, *p* < 0.0001, Figure 1A] and a significant time by diet interaction [*F*_(20,21)_ = 9.64, *p* < 0.0001, Figure 1A]. A trend toward a significant time by diet by genotype was also observed, but it did not quite reach statistical significance [*F*_(20,21)_ = 1.54, *p* = 0.063]. Although the KI animals gained significantly more weight over the 21-week period, there were no genotype differences in the weights of mice on either diet. When percent weight gain over the entire course of the experiment was evaluated in a two-way between subjects ANOVA with genotype and diet as factors, a main effect of diet was observed [*F*_(1,41)_ = 16.54, *p* < 0.001, Figure 1B]. A trend toward KI mice gaining a greater percent weight over time was also observed, but the diet by genotype interaction did not reach statistical significance (*p* = 0.061). Mice consumed significantly more HFD chow than SD chow [*F*_(1,41)_ = 10.48, *p* = 0.003, Figure 1C], but no significant genotype effect and no genotype by diet interaction were observed.

### Novel Open Field Test

**FIGURE 2:**
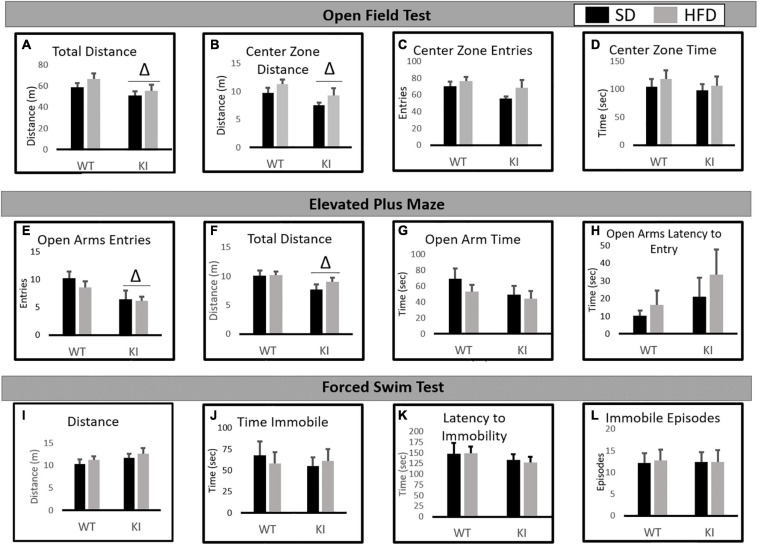
Behavioral consequences of chronic HFD on WT and KI mice. **(A)** The average total distance traveled in the open field for each group. **(B)** The average distance traveled in the center of the open field for each group. **(C)** The average number of entries into the center of the open field for each group. **(D)** The time spent in the center of the open field in each group. **(E)** The average number of open arm entries. **(F)** Total distance traveled in the elevated plus maze (EPM). **(G)** The average amount of time spent in the open arms. **(H)** The average latency until the first entry into the open arm. **(I)** The total distance traveled in the FST. **(J)** The time spent immobile in the FST. **(K)** The latency until the first immobile episode in the FST. **(L)** The number of immobile episodes in the FST. Results are expressed as the mean, and error bars indicate standard error of the mean. “Δ” indicates a main effect of genotype by two-way ANOVA, *p* < 0.05. For the open field and FST, *N* = 10 per WT group and 11 per KI group. For the EPM, *N* = 9 for the WT-SD, *N* = 10 for the KI-SD and the WT-HFD, and *N* = 11 for the KI-HFD group.

In the NOF, significant main effects of genotype were observed by two-way ANOVA for both the total distance traveled [*F*_(1,41)_ = 6.77, *p* = 0.0132, Figure 2A] and the distance traveled in the center [*F*_(1,41)_ = 5.76, *p* = 0.0214, Figure 2B], but no significant effect of diet and no diet by genotype interactions were observed for either measure. Slight trends toward increased center distance (*p* = 0.067) and total distance (*p* = 0.073) following HFD exposure were observed, but neither reached statistical significance. No significant effects of diet or genotype were observed for center entries (Figure 2C) or center time (Figure 2D).

### Elevated Plus Maze

In the EPM, KI mice entered the open arms less than WT mice [*F*_(1,39)_ = 7.82, *p* = 0.008, Figure 2E] and traveled less distance overall than WT animals [*F*_(1,39)_ = 5.26, *p* = 0.028, Figure 2F]. However, no significant effects of diet or genotype were observed on open arm time (Figure 2G) or the latency to enter the open arms (Figure 2H).

### Forced Swim Test

In the FST, no significant effects of diet or genotype were observed for distance traveled (Figure 2I), immobility time (Figure 2J), latency to immobility (Figure 2K), or the number of immobile episodes (Figure 2L).

### Hippocampal Gene Expression Analysis

**FIGURE 3:**
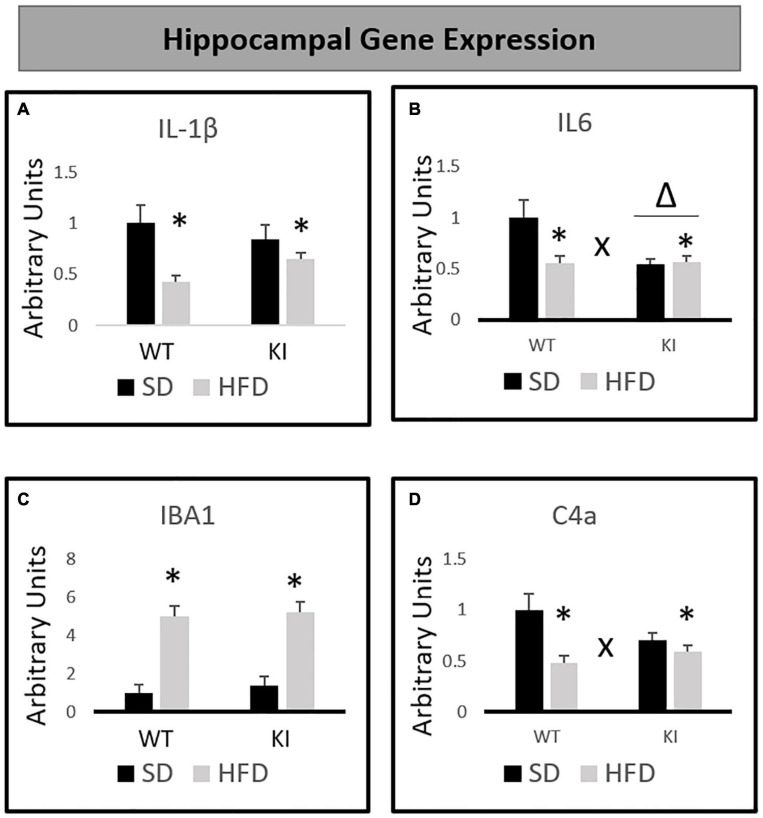
Effects of chronic HFD on the expression of inflammation-related genes in the hippocampus of WT and KI mice. **(A)** Hippocampal expression of interleukin-1β (IL-1β). **(B)** Hippocampal expression of interleukin-6 (IL-6). **(C)** Hippocampal expression of ionized calcium binding adaptor molecule 1 (IBA1). **(D)** Hippocampal expression of complement component 4a (C4a). All data are normalized to GAPDH. Results are expressed as the mean, and error bars indicate standard error of the mean. “Δ” indicates a main effect of genotype, “^∗^” indicates a main effect of diet, and “X” indicates a genotype by diet interaction by two-way ANOVA. *N* = 9 for the WT-SD, *N* = 10 for the WT-HFD group, and *N* = 11 for both KI groups.

Chronic exposure to HFD significantly reduced the expression of IL-1β [*F*_(1,40)_ = 11.55, *p* < 0.001, Figure 3A], but no significant effects of genotype and no genotype by diet interaction were observed for IL-1β. For IL-6, significant main effects of HFD [*F*_(1,40)_ = 5.63, *p* = 0.023, Figure 3B] and genotype [*F*_(1,40)_ = 6.35, *p* = 0.016, Figure 3B] were observed, as was a significant genotype by diet interaction [*F*_(1,40)_ = 6.83, *p* = 0.013, Figure 3B]. Overall, KI mice expressed less IL-6 in the hippocampus compared to WT mice, and HFD also led to a reduction in IL-6. However, the HFD-induced reduction in IL-6 was observed only in the WT mice, as evidenced by the significant interaction. HFD significantly increased the expression of IBA1 in both genotypes [*F*_(1,40)_ = 72.14, *p* < 0.001, Figure 3C], and no interaction or main effect of genotype was observed. For C4a, there was no significant effect of genotype. However, a significant main effect of HFD [*F*_(1,40)_ = 13.08, *p* = 0.001, Figure 3D] and a significant genotype by diet interaction [*F*_(1,40)_ = 5.80, *p* = 0.021, Figure 3D] were observed. Although HFD led to an overall reduction in C4a, this effect was only observed in the WT mice, as evidenced by the significant interaction.

**FIGURE 4:**
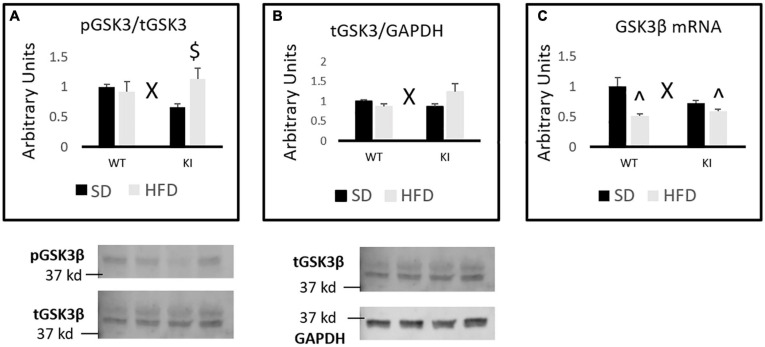
Effects of chronic HFD on GSK3β signaling in the hippocampus of WT and KI mice. **(A)** Quantification of western blots for phosphorylated GSK3β. Graph shows the ratio of phosphorylated GSK3β to total GSK3β. Representative image shown below. For the total GSK3β image, the lower band corresponds to GSK3β, while the upper band corresponds to GSK3α. **(B)** Quantification of western blots for total GSK3β compared to GAPDH. Graph shows the total GSK3β/GAPDH ratio. **(C)** Quantification of mRNA levels of GSK3β normalized to GAPDH. The results are expressed as the mean, and error bars indicate standard error of the mean. “X” indicates a significant genotype by diet interaction by two-way ANOVA, *p* < 0.05. “^” indicates significant difference compared to WT-SD and “$” indicates significant difference compared to KI-SD by Tukey’s *post hoc* test. *N* = 8 mice per group for all western blots. *N* = 9 for the WT-SD, *N* = 10 for the WT-HFD group, and *N* = 11 for both KI groups for C.

Chronic HFD led to genotype-dependent effects on the phosphorylation of GSK3β [genotype by diet interaction, *F*_(1,31)_ = 5.14, *p* = 0.031, Figure 4A]. Tukey’s *post hoc* tests indicate that HFD-fed KI mice exhibit an increased pGSK3β/tGSK3β ratio compared to KI mice fed SD, but no other individual group comparisons were significant. When total protein levels of GSK3β relative to GAPDH were assessed by western blot, a significant genotype by diet interaction was observed in which HFD tended to reduce GSK3β levels in WT mice but increase them in KI animals [*F*_(1,31)_ = 5.44, *p* = 0.027, Figure 4B]. However, Tukey’s *post hoc* test did not reveal any statistically significant differences between the groups (Figure 4B). When mRNA levels of GSK3β were measured, a significant genotype by diet interaction was again observed in which HFD reduced GSK3β mRNA levels more in WT mice than in KI animals [*F*_(1,40)_ = 6.45, *p* = 0.015, Figure 4C]. However, a significant main effect was also observed in which HFD significantly reduced GSK3β mRNA in the hippocampus overall [*F*_(1,40)_ = 19.83, *p* < 0.001, Figure 4C]. Tukey’s *post hoc* tests indicated that both of the HFD-fed groups had significantly less GSK3β mRNA than the WT-SD group.

## Discussion

In comparison to our previously published study that focused on males (Karth et al., 2019), the current results suggest that female mice are less sensitive than males overall to HFD-induced weight gain and HFD-induced alterations in the FST, EPM, and NOF. One limitation of both the current study and our previous work is that each study only evaluated a single sex. Thus, while we do report and discuss the different results we observed in the two studies, our ability to make meaningful statistical comparisons between the sexes would have been much improved had males and females been run in the same cohorts. We did our best to match the experimental conditions between the studies, but given the several years between studies and the different personnel involved in each set of studies, it is possible that there are some confounding variables impacting our results. Nonetheless, the HFD-fed female mice in this study did not exhibit obvious differences in weight gain compared to SD-fed mice until consuming HFD for more than 3 months. In contrast, male mice fed HFD exhibited greater weight gain than SD-fed controls after only 4–6 weeks (Karth et al., 2019). An earlier onset of weight gain in male compared to female c57BL6/J mice fed HFD has been reported previously (Hwang et al., 2010). This sex difference in weight gain has been suggested to result from a protective effect of estrogen against diet-induced obesity and has been shown to diminish with increasing age (Salinero et al., 2018). Our current results suggest that brain 5-HT deficiency increases the susceptibility of females to HFD-induced weight gain, but whether estrogen is involved in this effect was not established in the current study. Similarly, whether low levels of brain 5-HT would differentially impact HFD-induced weight gain in females at different time points throughout development remains to be determined.

In contrast to our previous report in males, in which HFD significantly reduced distance traveled in the center and entries into the center of the NOF (Karth et al., 2019), the current study found no such effects of HFD in females. However, in the current study, brain 5-HT deficiency reduced total distance traveled and distance traveled in the center of the NOF in females. In the corresponding study performed in males, brain 5-HT deficiency did not significantly impact locomotor activity in the NOF (Karth et al., 2019). Several of our other published studies have similarly reported no significant effects of brain 5-HT deficiency on distance traveled in the NOF in males (Sachs et al., 2013c, 2014). However, our studies with females (or both sexes evaluated together) have produced somewhat inconsistent results with respect to the effects of low 5-HT on NOF behavior. Indeed, one study that included both males and females (but did not evaluate sex differences) found a significant overall reduction in center distance in KI mice compared to WT (Sachs et al., 2013b), but a separate report examining males and females separately did not find any genotype differences in either sex (Sachs et al., 2014). The reasons for these discrepancies are not entirely clear, but it is important to note that the current work examines KI mice on a c57BL6/J background, where previous studies used animals on a mixed background. In addition, the experiments for each of the three studies were conducted in three different animal facilities on animals of different ages, and the estrous stage of female mice was not monitored in any of the studies.

The current results indicate that brain 5-HT deficiency reduces exploratory locomotion and open arm entries in the EPM in females. Our prior report in males also demonstrated that low brain 5-HT reduced total distance traveled in this test (Karth et al., 2019). Although the EPM is traditionally considered to measure anxiety-like behavior, several behavioral domains can contribute to EPM behavior. For example, the exploratory and locomotor drive of animals motivates an investigation of the entire maze, while the potential threats associated with the open arms counteract this drive. A study using factor analysis in rats has suggested that the primary factor driving EPM behavior may differ in males and females. Specifically, it has been reported that the behavior of male rats in the EPM is best explained by their anxiety levels (i.e., fear of potential threats that might be encountered in the open arms), whereas females tend to be driven primarily by overall locomotor drive in this test (Fernandes et al., 1999). The reasons for these reported sex differences are not clear, and it remains unknown whether similar results would be observed in mice. Although our results suggest that brain 5-HT deficiency leads to similar behavioral alterations in males and females in the EPM, whether the motivating factors that underlie this behavior is similar between the sexes has yet to be determined. Additional research in which the cognitive and behavioral alterations induced by HFD and brain 5-HT deficiency are directly compared in males and females would be required to resolve this issue.

Female mice tested in this study did not display any antidepressant-like responses to chronic HFD exposure in the FST regardless of genotype. In contrast, WT males fed a HFD were observed to display an antidepressant-like response in the FST whereas KI males on a HFD were not (Karth et al., 2019). While there is considerable debate regarding exactly what immobility in the FST reflects (de Kloet and Molendijk, 2016; Anyan and Amir, 2018), the test remains a potentially useful predictor of antidepressant-like effects. Nonetheless, additional studies that examine anhedonia or negative cognitive biases following HFD administration could provide more insight into the effects of HFD on “depression-like” behavior. It is also important to note that several of the previous studies documenting antidepressant-like effects of HFD observed these antidepressant-like effects only in animals in which “depression-like” phenotypes were induced by stress, not in control animals (Maniam and Morris, 2010a,b). While this was not necessary in males to observe antidepressant-like responses to HFD in the FST, it will be important to repeat the current studies in male and female mice that have been exposed to stress to determine the impact of low 5-HT on the ability of HFD to reverse stress-induced behavioral pathology. Of course, those types of studies could be partially confounded by sex- and 5-HT-deficiency-induced alterations in susceptibility to stress (Sachs et al., 2013b, 2014, 2015), but they could nonetheless provide useful information.

Our gene expression analyses also revealed strikingly different patterns of HFD-induced alterations in females than what we had previously reported in males (Karth et al., 2019). For example, in males, HFD was shown to significantly increase the expression of IL-1β (Karth et al., 2019). However in females, we observed a significant reduction in IL-1β mRNA expression in HFD-exposed animals compared to mice fed SD. We previously observed no significant effects of HFD on IL-6 or IBA1 expression in males, but HFD significantly increased IBA1 expression in both genotypes of females. Regarding IL-6, brain 5-HT deficiency led to a baseline reduction in IL-6 levels, and HFD reduced IL-6 levels in WT females, but not in KI females. Brain 5-HT deficiency appeared to blunt the effects of HFD on hippocampal C4a expression in both males and females. However, in males, HFD significantly increased C4a expression (Karth et al., 2019), but in females, HFD significantly reduced C4a expression in WT animals, but had no significant effect in KI mice. The reasons for these sex differences remain unclear. Overall, HFD appeared to have less of an effect on the body weight of females compared to males, which could potentially impact their cytokine levels compared to males. However, there are a number of other potential explanations for these sex differences. For example, gonadal hormones could be at least partially responsible, as estradiol is known to reduce IL-6 expression (Pottratz et al., 1994) and HFD has been shown to increase estrogen levels in female mice (Chakraborty et al., 2016). In addition, glucocorticoids are known to suppress the production of several cytokines, including IL-1β and IL-6 (Brattsand and Linden, 1996), and female rodents are well known to exhibit higher baseline levels of glucocorticoids than males and to exhibit many other differences in responses to stress hormones (Bangasser, 2013). Additional research would be required to determine the precise mechanisms underlying these sex differences in mRNA expression following HFD.

Similarly, the effects of chronic HFD and brain 5-HT deficiency on GSK3β signaling in the hippocampus were different in females than what we had previously reported in males. In males, HFD led to an increase in the pGSK3β/tGSK3β ratio in WT, but not KI, mice (Karth et al., 2019). In females, HFD increased the pGSK3β/tGSK3β ratio in KI, but not WT mice. There was a trend toward a reduced pGSK3β/tGSK3β ratio in SD-fed KI females compared to SD-fed WT females, but this effect did not reach significance in *post hoc* tests. In the initial description of Tph2(R439H)KI mice, KI animals were reported to have a reduced pGSK3β/tGSK3β ratio in the frontal cortex (but not hippocampus or striatum) compared to WT mice (Beaulieu et al., 2008), but the sex of mice was not reported in that study. The current results suggest that genetic brain 5-HT deficiency may differentially regulate GSK3β phosphorylation and protein expression in the hippocampus of females and males. In males, no significant effects of 5-HT deficiency or HFD were observed on the total protein levels of GSK3β in the hippocampus, but a significant genotype by diet interaction was observed in females in which total GSK3β tended to decrease following HFD in WT mice but increase in KI animals. The reasons for these sex differences remain unknown, but they could again be dependent on gonadal hormones, as estradiol has been shown to impact GSK3β phosphorylation and activity in cultured hippocampal neurons (Cardona-Gomez et al., 2004).

Although the current results revealed significant genotype by diet interactions for both the protein and mRNA levels of GSK3β, the nature of these interactions were not identical. HFD led to a significant reduction in GSK3β mRNA levels in WT mice despite inducing only a very slight (and not significant) reduction in protein levels of GSK3β in these animals. In KI mice, HFD tended to increase protein levels of GSK3β (although this did not quite reach significance) while having virtually no effect on GSK3β mRNA levels. These results suggest that the regulation of GSK3β by HFD and 5-HT is quite complex and could occur through multiple mechanisms. In addition, given that no significant effects of HFD or brain 5-HT were reported previously on the hippocampal mRNA levels of GSK3β in males, these results suggest that males and females exhibit distinct molecular adaptations in the hippocampus in response to chronic HFD exposure. The functional significance of these sex-specific molecular responses is not currently clear, and the mechanisms that lead to these sex differences remain to be elucidated. However, these sex differences may have important implications for hippocampus-dependent behaviors, and future research should continue to evaluate sex differences in HFD-induced alterations in additional types of behavioral processes. Given the importance of the hippocampus in processes such as learning, spatial memory, and pattern separation, future research should evaluate potentially sex-specific cognitive alterations induced by chronic exposure to HFD in mice with varying levels of brain 5-HT.

## Data Availability Statement

The raw data supporting the conclusions of this article will be made available by the authors, without undue reservation.

## Ethics Statement

The animal study was reviewed and approved by the Villanova University IACUC.

## Author Contributions

BS conceptualized and designed the study, wrote the first draft of the manuscript, and performed data analysis. SH, AW, and KB performed the experiments, collected and analyzed the data, and revised the manuscript. All authors contributed to the article and approved the submitted version.

## Conflict of Interest

The authors declare that the research was conducted in the absence of any commercial or financial relationships that could be construed as a potential conflict of interest.

## Supplementary Material

Supplementary Table 1 A list of primers used for real-time PCR.
