# Supplementary material for: The Effects of Brain Serotonin Deficiency on Responses to High Fat Diet in Female Mice
Source: Front Neurosci. 2021 Jul 2;15:683103. doi: 10.3389/fnins.2021.683103 (PMC8282998; doi:10.3389/fnins.2021.683103)
Supplement: Supplementary Table 1 — A list of primers used for real-time PCR. [file Presentation_1.PPTX]

## Slide 1
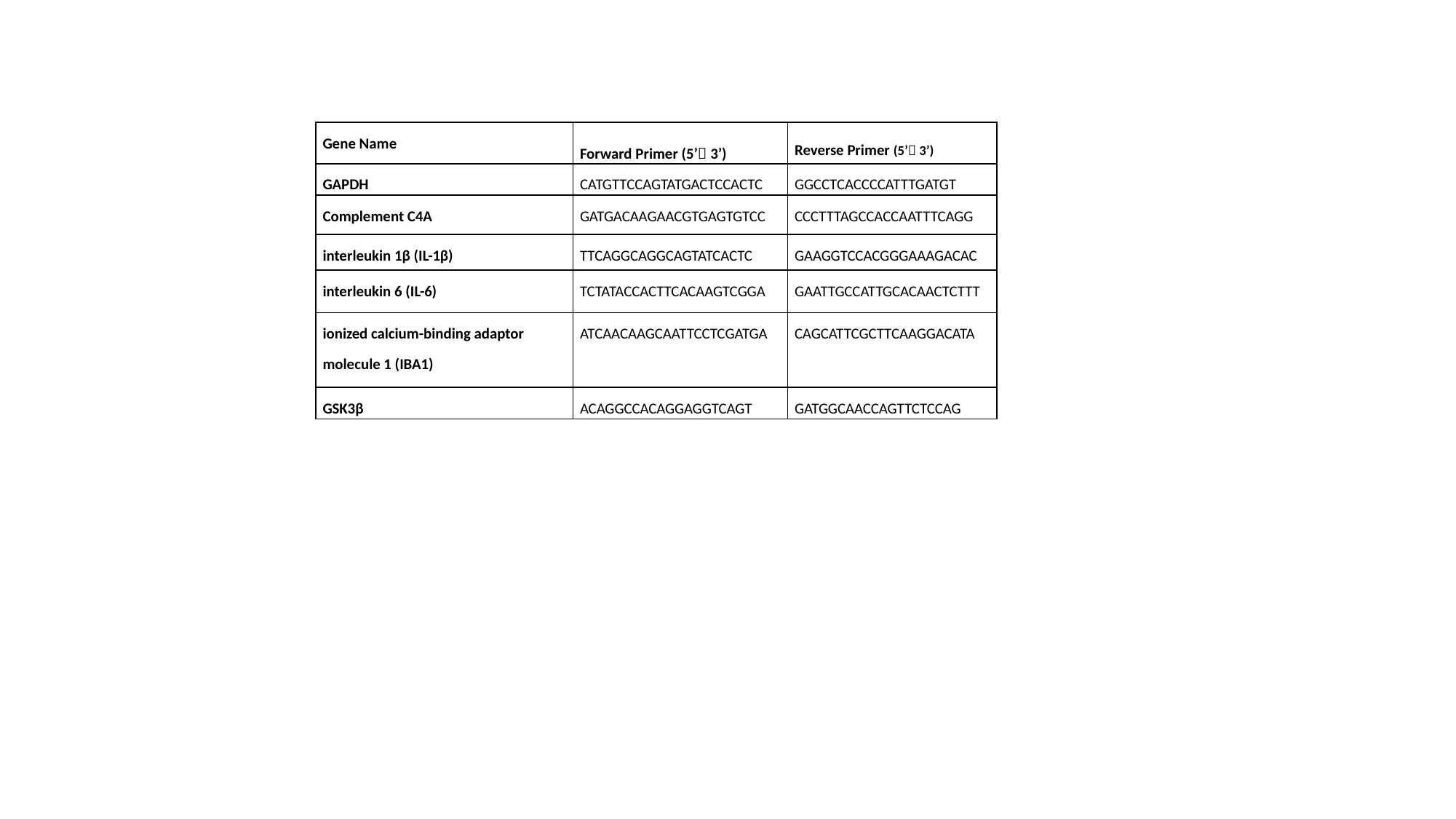

| Gene Name | Forward Primer (5’ 3’) | Reverse Primer (5’ 3’) |
| --- | --- | --- |
| GAPDH | CATGTTCCAGTATGACTCCACTC | GGCCTCACCCCATTTGATGT |
| Complement C4A | GATGACAAGAACGTGAGTGTCC | CCCTTTAGCCACCAATTTCAGG |
| interleukin 1β (IL-1β) | TTCAGGCAGGCAGTATCACTC | GAAGGTCCACGGGAAAGACAC |
| interleukin 6 (IL-6) | TCTATACCACTTCACAAGTCGGA | GAATTGCCATTGCACAACTCTTT |
| ionized calcium-binding adaptor molecule 1 (IBA1) | ATCAACAAGCAATTCCTCGATGA | CAGCATTCGCTTCAAGGACATA |
| GSK3β | ACAGGCCACAGGAGGTCAGT | GATGGCAACCAGTTCTCCAG |
